# Renal Infarction From Over-the-Counter Testosterone Booster Pills: A Case Report

**DOI:** 10.7759/cureus.37082

**Published:** 2023-04-03

**Authors:** Carlos Gaibor, Pablo Dayer

**Affiliations:** 1 Internal Medicine, St Luke's Hospital, Chesterfield, USA; 2 Nephrology, St. Luke's Hospital, Chesterfield, USA

**Keywords:** testosterone supplements, over-the-counter drugs, testosterone, renal artery infarction, renal infarction

## Abstract

Renal infarction is a challenging diagnosis that usually requires a high level of clinical suspicion because its clinical presentation is often attributed to more frequent causes. Here, we present the case of a young male with right flank pain. A computed tomography (CT) of the abdomen ruled out nephrolithiasis; hence, a CT urogram was performed, which revealed an acute right kidney infarction. The patient had no personal or family history of clotting disorders. Subsequent tests for atrial fibrillation, an intracardiac shunt, and genetic causes were negative, and a presumptive diagnosis of a hypercoagulable state from over-the-counter testosterone supplements was made.

## Introduction

This case presents a renal infarction caused by a hypercoagulable state due to over-the-counter testosterone booster pills. This disease is often an underreported and overlooked diagnosis [1]. The two major causes are thromboembolic, usually originating from the heart or the aorta, and in situ thrombosis, due to an underlying hypercoagulable state, injury, or dissection of the renal artery [2]. Cardiogenic and hypercoagulable etiologies are more prevalent in older individuals than in younger patients, in whom renal artery injury is more prevalent [3]. Furthermore, patients usually complain of acute onset of flank or abdominal pain frequently accompanied by nausea, vomiting, and occasionally a fever mimicking more common conditions such as renal colic or acute pyelonephritis [4]. A computed tomography (CT) without contrast is the preferred initial test because it could also rule out more common causes [5]. If the clinical suspicion remains high, a contrast-enhanced CT or an MRI with gadolinium should be considered, depending on renal function [4]. In terms of treatment, there is no general consensus, with multiple approaches proposed that include anticoagulation, percutaneous endovascular therapy (thrombolysis, thrombectomy with or without angioplasty, or stent placement), and open surgery [5].

## Case presentation

**Figure 1:**
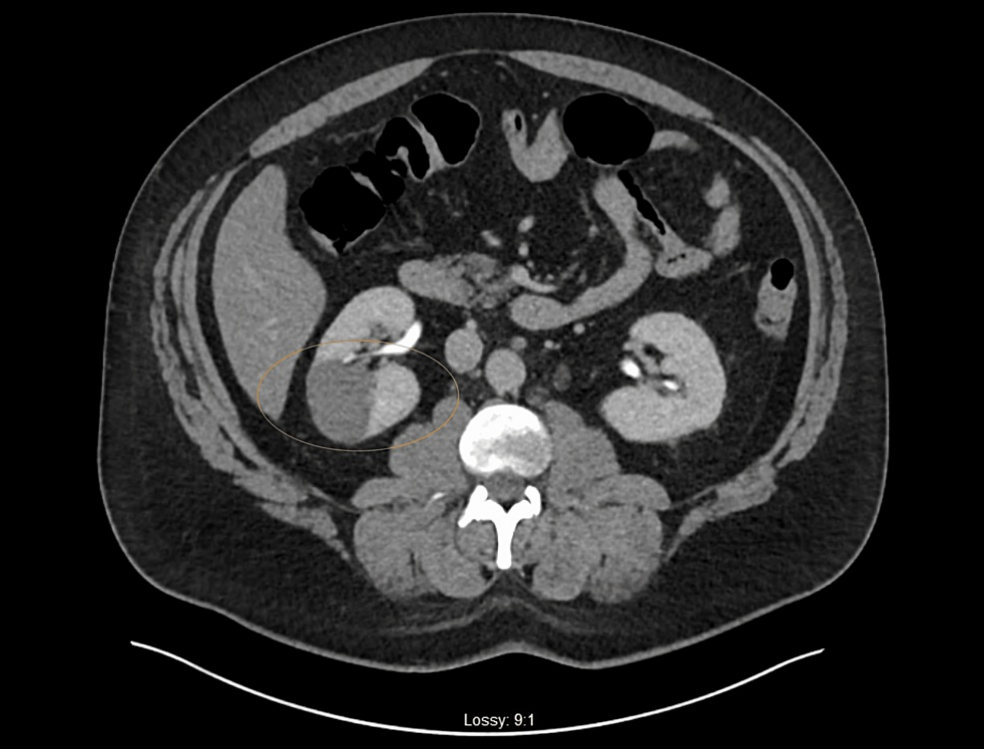
CT urogram showing right renal infarction

The patient was a 42-year-old male who had been consuming over-the-counter testosterone booster supplements for the past six months and presented with acute onset right flank abdominal pain. The patient had a significant past medical history for hypertension (HTN), hyperlipidemia (HLD), obstructive sleep apnea (OSA), anxiety, gastroesophageal reflux disease (GERD), and fatty liver disease, but no familial history of hypercoagulable disorder. The pain was constant and severe, located in the right flank, radiated to the right lower abdomen, and was associated with nausea and vomiting. The patient denied having a fever, shortness of breath, chest pain, dysuria, hematuria, or a recent history of abdominal trauma. In the emergency department, computed tomography (CT) of the abdomen without contrast ruled out urolithiasis, and urinalysis (UA) results were unremarkable. Consequently, the patient was discharged with hydrocodone and acetaminophen tablets for pain control. However, the patient returned because his pain had worsened despite the medication prescribed. As a result, a CT urogram with contrast was performed, which revealed an acute right kidney infarction involving the superior branch of the right renal artery (Figure 1).

The initial laboratory results were relevant for hemoglobin of 15.1 g/dl, blood urea nitrogen of 12 mg/dl, and creatinine of 1.0 mg/dl (Table 1).

**Table 1:** The patient's initial laboratory results MCV: mean corpuscular volume; MCH: mean corpuscular hemoglobin; MCHC: mean corpuscular hemoglobin concentration; RDW: red cell distribution width; HCO3: bicarbonate; BUN: blood urea nitrogen; AST: aspartate aminotransferase; ALT: alanine transaminase

Laboratory	Result	Normal range
White blood cells	9.5 K/uL	4.3 - 10.0 K/uL
Hemoglobin	15.1 gr/dl	13.6-16.5 gr/dl
Hematocrit	44.20%	40.o -48.0 %
MCV	94.0 fL	82.0-99.0 fL
MCH	32.1 pg	27.2-32.6 pg
MCHC	34.2 gr/dl	31.5-35.5 gr/dl
RDW	12.60%	11.5-14.5 %
Sodium	134 mmol/L	137-145 mmol/L
Potassium	3.9 mmol/L	3.5-4.9 mmol/L
Chloride	99 mmol/L	98-107 mmol/L
HCO3	25 mmol/L	22-30 mmol/L
BUN	12 mg/dl	9-20 mg/dl
Creatinine	1.0 mg/dl	0.7-1.3 mg/dl
Glucose	126 mg/dl	74-106 mg/dl
Calcium	9.2 mg/dl	8.4-10.2 mg/dl
Protein total	8.1 gr/dl	6.5-8.6 gr/dl
Albumin	4.5 gr/dl	3.5-5.0 gr/dl
Alkaline phosphatase	6.3 U/L	38-126 U/L
Bilirubin, total	0.5 mg/dl	0.2-1.3 mg/dl
AST	88 U/L	14-54 U/L
ALT	111 U/L	<= 50 U/L
Lipase	79 U/L	23-300 U/L

**Figure 2:**
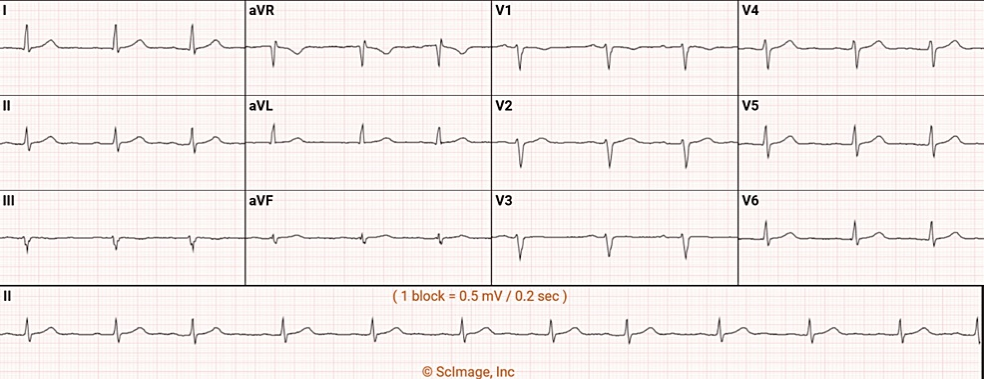
The electrocardiogram showed a normal sinus rhythm with a few premature ventricular contractions

The electrocardiogram showed a normal sinus rhythm with a few premature ventricular contractions (Figure 2).

Furthermore, telemetry monitoring did not report atrial fibrillation, and transthoracic echocardiography with a bubble study did not show an ejection fraction of 68% without any evidence of an atrial or ventricular shunt or an intracardiac thrombus. Subsequently, vascular surgery and nephrology departments were consulted, and a heparin drip, intravenous fluids, and intravenous morphine were commenced with no indication for revascularization because the pain started more than 24 hours earlier. A CT angiogram was not done given the unnecessary exposure to intravenous contrast that could affect renal function. The anticoagulation workup was negative, including for antinuclear antibodies (ANA); beta-2 glycoprotein; immunoglobulin G, A, and M; cardiolipin; lupus anticoagulant; factor II; factor V Leiden mutation; antithrombin III activity; protein S free and total; protein S functional activity; and protein C activity. Testosterone levels were found low at 102 ng/dl (normal range: 132-813 ng/dl), and the patient was educated on avoiding these supplements and discharged with Eliquis to be taken for six months.

## Discussion

The patient was a 42-year-old muscular male without a significant personal or familial medical history of clotting disorders who was diagnosed with acute right renal infarction. Cardiac etiology was ruled out with negative telemetry monitoring for atrial fibrillation or any other type of arrhythmia and unremarkable transthoracic echocardiography for intracardiac shunts or thrombus. Similarly, the coagulation workup was inconspicuous, and additional etiologies were therefore considered, including a hypercoagulable state caused by an exogenous substance. Subsequently, the patient admitted to consuming over-the-counter testosterone booster pills that are associated with low testosterone levels, and the muscular body of the patient suggested suppression of endogenous testosterone production by the consumption of over-the-counter supplements that are a known risk for a hypercoagulable state and thromboembolic risk [6-7]. As a result, this case indicates the possibility of an over-the-counter testosterone booster in the development of this patient's hypercoagulable state and, in turn, a thromboembolic risk causing renal infarction in this patient. For this reason, this case presents the importance of avoiding over-the-counter supplements, especially without medical supervision, as well as the importance of further research regarding the adverse effects of testosterone as a contributor to hypercoagulable states.

## Conclusions

This case highlights the importance of taking a complete history and asking the patient about both prescribed and over-the-counter medications or supplements. Patients may not view over-the-counter medications as potentially hazardous, but testosterone, like nonsteroidal anti-inflammatory drugs (NSAIDs), can carry serious side effects. It is imperative that both marketers and healthcare providers warn patients about a potential hypercoagulable state from testosterone to avoid the dreadful outcome seen in this patient.
